# Editorial: Novel theragnostic approaches in thoracic malignancies

**DOI:** 10.3389/fonc.2026.2006660

**Published:** 2026-09-30

**Authors:** Andrea Cimini, Francesca Russo, Maria Silvia De Feo, Habibollah Dadgar

**Affiliations:** 1 Nuclear Medicine Unit, St. Salvatore Hospital, L’Aquila, Italy; 2 Department of Radiological Sciences Oncology and Anatomo-Pathology, Universita degli Studi di Roma La Sapienza, Rome, Italy; 3 Cancer Research Center, Imam Reza International University, Razavi Hospital, Mashhad, Iran

**Keywords:** imaging, lung cancer, theragnostic, therapy, thoracic malignancies

Thoracic malignancies represent a heterogeneous group of diseases characterized by complex biological behavior, substantial inter- and intratumoral heterogeneity, and a rapidly evolving diagnostic and therapeutic landscape. In recent years, advances in medical imaging, molecular profiling, targeted therapies, immunotherapy, and minimally invasive interventions have progressively contributed to a more personalized approach to thoracic oncology. In parallel, the increasing availability of quantitative imaging and biological biomarkers is creating new opportunities for more accurate disease characterization and patient stratification.

The Research Topic “Novel Theragnostic Approaches in Thoracic Malignancies” brings together 12 contributions addressing several aspects of contemporary thoracic oncology, including advanced imaging, radiomics, prognostic biomarkers, image-guided interventions, molecularly targeted treatments, immunotherapy, surgery, and innovative drug-delivery strategies. Although most of the contributions do not address theragnostics in the strict sense, they collectively illustrate the progressive convergence of diagnostic characterization and therapeutic decision-making that underlies the development of this paradigm.

Several contributions focus on the potential of advanced imaging to improve the characterization and stratification of lung cancer. Fang et al. developed an arterial-phase CT-based habitat imaging model for the non-invasive prediction of PD-L1 expression in NSCLC. The habitat imaging model outperformed whole-tumor radiomics, while the combination of radiomics and clinical variables achieved the highest predictive performance, suggesting a potential role for quantitative CT in the pre-immunotherapy assessment of patients. Dong et al. similarly investigated CT-based habitat radiomics for the preoperative differentiation of adenocarcinoma in situ/minimally invasive adenocarcinoma from invasive adenocarcinoma presenting as ground-glass nodules. Their combined model achieved an AUC of 0.897 in the external validation cohort, supporting the potential value of intratumoral heterogeneity assessment for preoperative characterization.

The prognostic role of imaging was addressed by Murakami et al., who developed a preoperative risk scoring system for patients with clinical stage IB lung adenocarcinoma. Smoking history, serum CEA levels, and tumor SUVmax on PET were independently associated with overall survival and were incorporated into a simple risk score for preoperative risk stratification. Beyond lung cancer, Chen et al. investigated the tumor immune microenvironment in thymic epithelial tumors and found distinct prognostic associations for CD20-positive and CD204-positive tumor-infiltrating immune cells, highlighting the potential relevance of immune-cell composition for risk stratification in these rare malignancies.

The Research Topic also includes several contributions addressing innovative therapeutic strategies. Chen et al. evaluated CT-guided ^125^I radioactive seed implantation in patients with advanced NSCLC who had progressed after radiotherapy or chemotherapy. The treatment was associated with a median overall survival of 16 months, together with improvements in quality-of-life measures and changes in peripheral immune-cell populations during follow-up. Wu and Zhang reported a long-term case of tracheal adenoid cystic carcinoma treated with repeated bronchoscopic interventions, including cryotherapy, thermal ablation, mechanical debulking, and para-toluenesulfonamide injection, illustrating the potential role of individualized endoscopic treatment in patients with complex central airway disease.

Molecularly guided treatment was represented by two case reports. Liu et al. described complete remission of an early-stage NSCLC harboring the rare compound EGFR G719C and S768I mutations following treatment with furmonertinib, with subsequent surgical resection showing no residual viable tumor. Flauto et al. reported a thoracic inflammatory myofibroblastic tumor harboring a rare TPR-ALK fusion. After an initial response followed by progression during crizotinib treatment, subsequent lorlatinib therapy resulted in marked tumor regression and sustained radiological disease control at 150 days. These cases underscore the importance of comprehensive molecular characterization and treatment adaptation according to actionable genomic alterations and disease evolution.

Other contributions further illustrate the complexity of individualized treatment. Huang and Li reported the successful rechallenge with the PD-1/CTLA-4 bispecific antibody QL1706 in a patient with advanced pulmonary squamous cell carcinoma complicated by multidrug-resistant tuberculosis. Following stabilization of tuberculosis, the patient achieved approximately 11 months of progression-free survival without tuberculosis reactivation or grade ≥3 immune-related adverse events. Sharif et al. reviewed direct pulmonary delivery strategies for siRNA therapeutics, emphasizing inhaled and bronchoscopic approaches as potential means of increasing local drug exposure while limiting systemic toxicity and discussing the challenges that remain for clinical translation.

Finally, Qi et al. investigated a modified same-intercostal chest tube placement technique after uniportal VATS lobectomy and found lower rates of moderate-to-severe acute postoperative pain compared with conventional placement. Zhang et al. described the management of a 15-year-old patient with minimally invasive lung adenocarcinoma and reviewed reported cases of lung cancer in patients aged ≤18 years, highlighting the diagnostic and therapeutic challenges of this uncommon population. Although these contributions are not directly related to theragnostics, they further emphasize the importance of individualized decision-making across different stages of thoracic cancer management.

Taken together, these studies illustrate two complementary dimensions of modern thoracic oncology. On the one hand, advanced imaging, radiomics, molecular profiling, and characterization of the tumor microenvironment are providing increasingly detailed information about tumor phenotype and biological behavior. On the other hand, targeted therapies, image-guided interventions, immunotherapy, and innovative drug-delivery systems are expanding the therapeutic possibilities available to selected patients. The next challenge is to integrate these two dimensions.

This is where the concept of theragnostics becomes particularly relevant. Traditionally developed in nuclear medicine through the combination of molecular imaging and targeted radionuclide therapy, theragnostics is based on the principle that the biological information used to identify and characterize a target can also guide its treatment. In thoracic oncology, this concept may extend beyond radiopharmaceutical therapy to include the integration of functional and molecular imaging, quantitative biomarkers, targeted treatment, image-guided intervention, and treatment-response assessment.

Within this Research Topic, the study by Chen et al. on CT-guided ^125^I seed implantation most directly exemplifies this concept. In this approach, imaging is not simply used for diagnosis or staging, but directly contributes to treatment planning and accurate delivery of radioactive seeds to the target lesion. The diagnostic and therapeutic components are therefore integrated within the same clinical pathway.

More broadly, the other contributions can be viewed as important building blocks for the future development of theragnostic strategies. Quantitative imaging may identify imaging phenotypes associated with specific molecular or immunological characteristics; molecular profiling may identify actionable targets; and image-guided techniques may enable increasingly precise local treatment. The integration of these approaches could ultimately allow treatment to be selected and adapted according to the specific biological and spatial characteristics of each tumor.

Several challenges, however, remain before this vision can be fully translated into clinical practice. Many of the strategies presented in this Research Topic are currently supported by retrospective studies, relatively small cohorts, or individual case reports: in this context, prospective validation, standardized biomarkers, reproducibility across institutions, and well-designed clinical trials will therefore be essential. At the same time, technological innovation should ultimately be accompanied by demonstrated clinical benefit, appropriate patient selection, and careful assessment of safety and quality of life.

In conclusion, the contributions collected in this Research Topic provide a broad overview of the rapidly evolving landscape of thoracic oncology, spanning advanced imaging, biological characterization, molecularly targeted therapies, immunotherapy, image-guided interventions, and innovative treatment strategies. While only some of these approaches can be considered theragnostic in the strict sense, together they illustrate the progressive convergence between diagnosis and treatment. Future advances in thoracic oncology will likely depend increasingly on this integration, moving from improved characterization of disease toward truly individualized strategies in which diagnostic information directly informs therapeutic selection, delivery, and monitoring.

